# Unveiling the role of BON domain-containing proteins in antibiotic resistance

**DOI:** 10.3389/fmicb.2024.1518045

**Published:** 2025-01-07

**Authors:** Shengwei Sun, Jinju Chen

**Affiliations:** ^1^Department of Fibre and Polymer Technology, School of Engineering Sciences in Chemistry, Biotechnology and Health, KTH Royal Institute of Technology, Stockholm, Sweden; ^2^Department of Materials, Loughborough University, Loughborough, United Kingdom

**Keywords:** cell envelope, BON domain-containing protein, antibiotic resistance, structure and function, pathogens

## Abstract

The alarming rise of antibiotic-resistant Gram-negative bacteria poses a global health crisis. Their unique outer membrane restricts antibiotic access. While diffusion porins are well-studied, the role of BON domain-containing proteins (BDCPs) in resistance remains unexplored. We analyze protein databases, revealing widespread BDCP distribution across environmental bacteria. We further describe their conserved core domain structure, a key for understanding antibiotic transport. Elucidating the genetic and biochemical basis of BDCPs offers a novel target to combat antibiotic resistance and restore bacterial susceptibility to antibiotics.

## Introduction

The rapid emergence of antibiotic-resistant bacteria poses a significant public health threat, with infections causing increasing mortality across all age groups (Barlow, 2018; Murray et al., 2022; Akram et al., 2023). This challenge is particularly pronounced for Gram-negative pathogens, which exhibit a growing prevalence of resistance mechanisms compared to Gram-positive bacteria (Cornaglia and Rossolini, 2009; Pulingam et al., 2022). Notably, Gram-negative bacteria (GNB) are associated with higher mortality in critically ill patients due to their intrinsic resistance to many antibiotics (Li et al., 2024). This multi-layered resistance often involves complex mechanisms hindering antibiotic action (Al-Hasan, 2021; Saxena et al., 2023). Therefore, elucidating the molecular pathways underlying antibiotic resistance in Gram-negative bacteria is crucial to address this unmet medical need.

Unlike Gram-positive bacteria, all GNB are surrounded by a unique cell outer membrane (OM). In contrast to the cytoplasmic membrane, the OM of GNB is highly asymmetric, with phospholipids in the inner leaflet and a glycolipid known as lipopolysaccharide (LPS) in the outer leaflet, and a number of proteins that are either anchored in or embedded into the plasma membrane, providing an additional layer of protection when exposed to antibiotics (Sun et al., 2022). Outer membrane proteins (OMPs) are the major protein components and represent ~50% of the outer membrane mass in GNB (Thoma et al., 2018). By now, almost all crystallized OMPs are built on a β-barrel assembly machinery (Noinaj et al., 2017). For example, the diffusion porins in *Escherichia coli*, such as OmpF, OmpC, and PhoE, are trimers of 16-stranded β-barrels (Vergalli et al., 2020). The β-barrel protein channels act as size exclusion channels and are responsible for the stringent permeability barrier restricting the entry of antibiotics (Delcour, 2009; Saxena et al., 2023). This is a general molecular mechanism of OMPs-mediated bacterial antibiotic resistance, which has been extensively explored in previous studies (Delcour, 2009; Choi and Lee, 2019; Ude et al., 2021). However, this is not the case for BON (bacterial OsmY and nodulation) domain-containing protein (BDCP), a ubiquitous OMP across multiple bacterial species.

BON is a binding domain, lacks conserved residues that indicate enzyme active sites, and plays an active role in the interaction with bacterial phospholipid membranes (Yeats and Bateman, 2003). Supporting the role of BDCPs in antibiotic resistance, recent studies have shown their involvement in this critical process. For instance, the pathogen *Acinetobacter baumannii*, known for its dual-BON domain protein, exhibits resistance to the lipooligosaccharide (LOS)-binding antibiotic polymyxin (Henry et al., 2012). A protein, namely BonA, played a vital role in the adaption of *A. baumannii* outer envelope to the effects of polymyxin (Grinter et al., 2021). DolP, formerly YraP, is a dual BON-domain lipoprotein found in *E. coli* and other GNB (Bryant et al., 2020). Loss of DolP resulted in the disruption of OM integrity, and induced increased susceptibility to antibiotics (e.g., vancomycin) (Bos et al., 2014; Morris Faye et al., 2018; Ranava et al., 2021). LysM domain BON family protein was found to have a binding affinity for carbapenem antibiotics, rendering them unable to bind to their targets in *E. coli* and clinical isolates (Ali et al., 2020). A recent study identified a novel BON domain-containing protein (BDCP) from an unculturable bacterium exhibiting efflux pump activity. This BDCP confers bacterial resistance to a broad spectrum of antibiotics, particularly ceftazidime (Sun et al., 2023). Despite their potential role in antibiotic resistance, the contribution of BDCPs has been largely overlooked. This gap in knowledge hinders our ability to address the escalating crisis of antibiotic-resistant bacteria. Moving forward, a deeper understanding of BDCP-mediated resistance mechanisms is essential to combat this critical public health threat.

In this perspective, we will discuss the widespread distribution and function of BON domain-containing proteins (BDCPs) across diverse bacterial species. We aim to elucidate the structure-function relationship of BDCPs and their potential role in mediating antibiotic resistance in specific pathogens. By deciphering their critical roles in antibiotic resistance development, BDCPs emerge as a promising target for novel therapeutic strategies. This approach could enhance bacterial susceptibility to existing antibiotics and pave the way for innovative clinical interventions for prevention and treatment.

## Widespread distribution of BDCP

**Figure 1:**
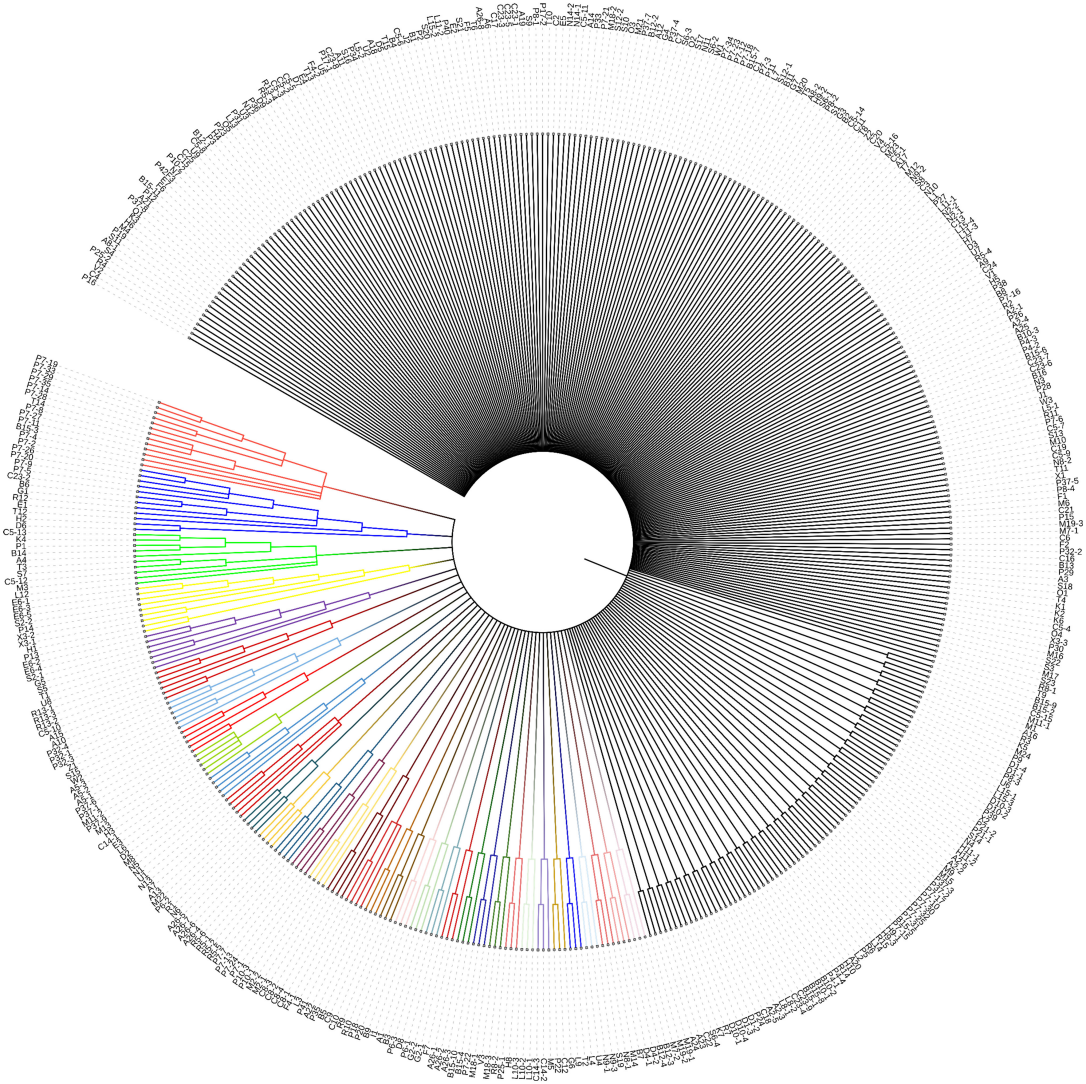
Phylogenetic tree analysis of various sources of BDCP. These 463 protein sequences were aligned using MUSCLE implemented in the phylogenetic analysis program MEGA-X (Kumar et al., 2018), which was subsequently used as the input for constructing a neighbor-joining phylogenetic tree to infer evolutionary relationships for these protein families. Different letters with different numbers (e.g., A1, A2, A3, B1, B2, and B3) on the clade represent different sources of bacteria (see Supplementary Excel form). The same letters with different numbers (e.g., A1-1, A1-2, and A1-3) represent that they come from the same genus but different species.

Unlike many well-characterized outer membrane proteins (OMPs) in Gram-negative bacteria (GNB), BON domain-containing proteins (BDCPs) remain relatively understudied. This gap in knowledge exists despite their potential role in antibiotic resistance. To investigate their prevalence, we search several protein databases including the National Center for Biotechnology Information (NCBI), UniProt, and RCSB Protein Data Bank (RCSB PDB) using the keyword “BON domain-containing protein.” In the NCBI database records, there are 102,824 results of BDCPs identified from bacteria, for example, some pathogens with amazing numbers are *Pseudomonas aeruginosa* (5739), *Klebsiella pneumoniae* (3671), *Acinetobacter baumannii* (2504), *Burkholderia multivorans* (2107), *Vibrio parahaemolyticus* (1442), and *Legionella pneumophila* (1435). Based on the result from the UniProt database, 51,284 BDCPs are identified by their accession numbers, organism names, and key features. The RCSB PDB provides only one example with 3D protein structure, namely BonA, from *Acinetobacter baumannii*. Given NCBI database contains a large number of redundant protein sequences, we select plenty of BDCP from the Uniprot database where a large resource of protein sequences, and associated detailed annotations are included (The UniProt, 2023). In the Uniprot knowledgebase, BDCPs were likewise found in a variety of clinical pathogens (numbers) such as *E. coli* (178), *Pseudomonas aeruginosa* (84), *Klebsiella pneumoniae* (69), *Acinetobacter baumannii* (43), *Burkholderia multivorans* (50), *Vibrio parahaemolyticus* (19), *Legionella pneumophila* (46), *Burkholderia cenocepacia* (99), *Burkholderia pseudomallei* (44), *Sinorhizobium meliloti* (32), and *Salmonella enterica* (727). The top 1,000 protein sequences from a huge diversity of bacteria were picked up and used for the next analysis. After removing some redundant, repetitive, and unreviewed protein sequences, 463 different sequences were ultimately adopted to construct a phylogenetic tree (Figure 1). It showed that a substantial source of bacteria displayed considerable variation in their distribution across bacterial types and names, including those from both culturable and unculturable bacteria (e.g., U3–U7) and involving strain names ranging from “A” to “Z” (see Supplementary Excel form). The length of amino acids varies greatly, even among strains from the same genus and species. It was found that about half of these 463 proteins can be aggregated into clusters even though these BDCPs were from different strain families, with branches consisting of 2–13 proteins. Several previously reported BDCPs such as DolP and OsmY from *E. coli* (shown as E6-1-E6-5 in the tree) were assigned into one clade (No. 4 [in yellow] and No. 5 [in purple]). These figures provide crucial evidence showing that BDCPs are widely distributed in diverse environmental bacteria, especially those notorious pathogens, but their roles in antibiotic resistance development have received little attention and lack of significant research, which is a major knowledge gap in current clinical and environmental pathology. Meta-genomic datasets provide valuable insights into protein diversity by directly extracting the total DNA from various environmental samples, including soil, water, sediment, and extreme environments such as terrestrial hot springs and deep-sea hydrothermal vents. Using bioinformatic analysis, such as open reading frame (ORF) finder search (https://www.ncbi.nlm.nih.gov/orffinder/), protein BLAST (BlastP) (https://blast.ncbi.nlm.nih.gov/Blast.cgi?PAGE=Proteins) and phylogenetic analysis, numerous hypothetical proteins with homology to reported BDCPs have been identified (Sun et al., 2023). These newly identified BDCP genes can then be heterologously expressed in bacterial or yeast systems for functional characterization, including *in vitro* and *in vivo* activity studies. Such approaches enhance our understanding of the distribution of BDCPs across diverse environments and their potential role in the development of antibiotic resistance.

## Structural diversity of BDCP across the pathogens

Recent advancements in structural biology have revolutionized our ability to understand protein. High-resolution structures can now be obtained for macromolecules, revealing their dynamics and interactions in unprecedented detail. Coupled with breakthroughs in artificial intelligence (AI) like AlphaFold (Jumper et al., 2021) and ever-increasing computational power, we are now achieving a level of accuracy and predictability in protein structure and function analysis (Aithani et al., 2023; Khanppnavar et al., 2024) that was previously unimaginable.

**Figure 2:**
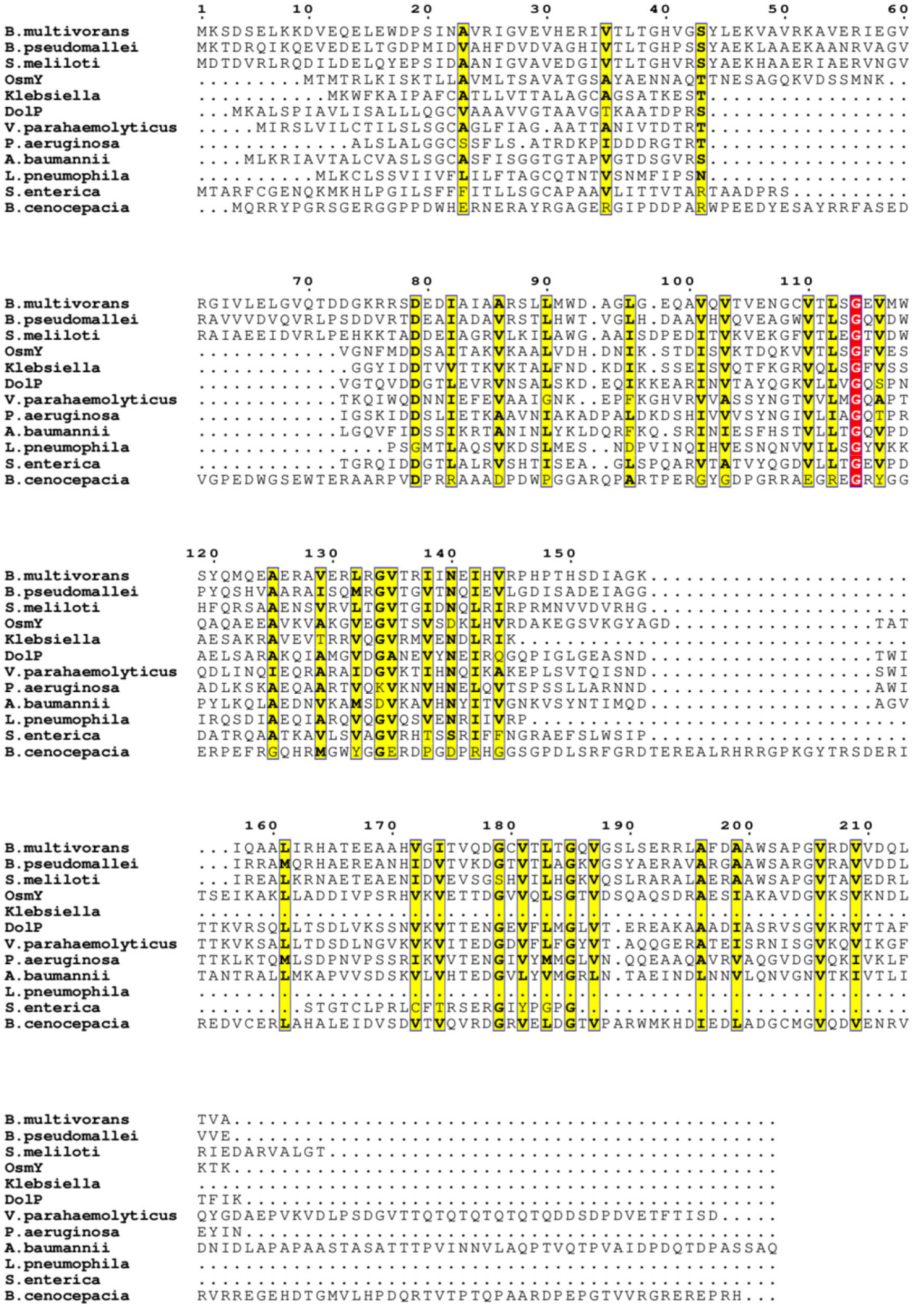
Multiple sequence alignment of these BDCPs from various pathogens. Similar residues are written with black bold characters and boxed in yellow. The same residues are written with white characters and boxed in red background.

**Figure 3:**
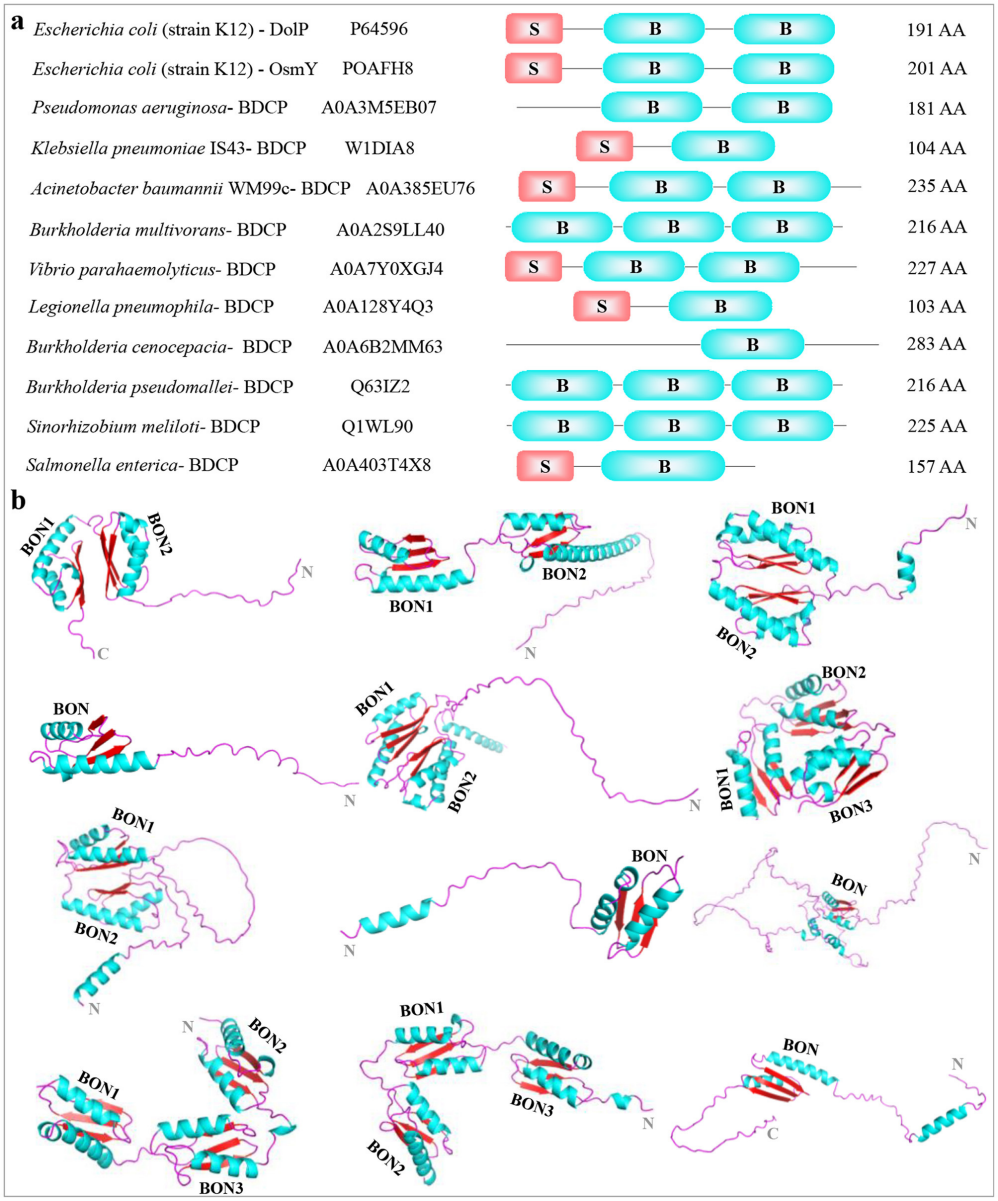
Display and comparison among different BDCPs identified from pathogenic bacteria. **(A)** Macroscopic comparison between BDCPs from several pathogens. The strain name, Uniprot entry number, schematic diagram of BDCPs (S, signal peptide; B, BON domain), and amino acid number were shown from left to right. **(B)** 3D structures of these above-mentioned BDCPs in pathogens. They were modeled using AlphaFold and consisted of α-helix (blue) the β-sheet (red), and a random coil (purple). The 3D structures of proteins (from left to right in each row) were collected from *Escherichia coli, Pseudomonas aeruginosa, Klebsiella pneumoniae, Acinetobacter baumannii, Burkholderia multivorans, Vibrio parahaemolyticus, Legionella pneumophila, Burkholderia cenocepacia, Burkholderia pseudomallei, Sinorhizobium meliloti*, and *Salmonella enterica*.

To explore the structural diversity and architectures of BDCP systems, we modeled, analyzed, and compared a panel of BDCPs from various bacterial pathogens including *E. coli, Pseudomonas aeruginosa, Klebsiella pneumoniae, Acinetobacter baumannii, Burkholderia multivorans, Vibrio parahaemolyticus, Legionella pneumophila, Burkholderia cenocepacia, Burkholderia pseudomallei, Sinorhizobium meliloti*, and *Salmonella enterica*. Multiple sequence alignment identified several conserved residues within the BDCPs from these bacterial species (Figure 2). Protein sequence analysis revealed significant variation in the lengths of BDCPs, reflecting the potential functional diversity of these proteins (Figure 3A). Notably, most BDCPs possess an N-terminal Sec signal peptide of ~20 residues. Interestingly, the number of BON domains varies considerably, with some pathogens like *B. multivorans, B. pseudomallei*, and *S. meliloti* harboring up to three domains.

AlphaFold server was then employed to predict the 3D structure of the BDCPs based on their amino acid sequences (Figure 3B). The overall architecture of these BDCPs appears to be relatively simple, primarily consisting of a combination of α-helix, β-sheet, and intrinsically disordered regions (IDRs) often located at the N- and C-termini (commonly referred to as random coils). Notably, the BON domain displayed a conserved fold across all analyzed sequences, consistently comprising two α-helixes and three β-sheets. This observation aligns well with the previously determined structure of a crystallized BonA protein using techniques including X-ray crystallography, electron microscopy, multiangle light scattering, and small-angle X-ray scattering (Grinter et al., 2021). It is worth noting that the random coil at the N- or/and C-terminal accounts for a large proportion in some structures, especially those that only have one BON domain, such as BDCP from *B. cenocepacia*. Hydrophobicity analysis indicated that several hydrophobic regions were located at the N-terminal extension and adjacent to the BON domain, which might be involved in the interaction between BDCPs and the cell membrane components such as phospholipids (Supplementary Figure S1) (Bryant et al., 2020). Additionally, residues located within hydrophobic pockets between adjacent BON domains, or even spanning three domains, might contribute to hydrophobic interactions that stabilize the over structure.

While BDCPs exhibit a distinct signature compared to typical β-barrel channel OMPs in GNB – a variable number of BON domains and significant intrinsically disordered regions (IDRs) at the termini – they share some functional similarities. Both contribute to cell envelope integrity and bacterial antibiotic resistance. Various pathogenic bacteria have evolved resistance to the available antibiotics in clinics, and multidrug-resistant bacteria have resulted in quite prevalent community-acquired infections (MacLean and San Millan, 2019). Here the identified pathogens including *E. coli, P. aeruginosa, K. pneumoniae, A. baumannii, B. multivorans, V. parahaemolyticus, L. pneumophila, B. cenocepacia, B. pseudomallei, S. meliloti*, and *S. enterica* represent deadly bacteria responsible for a wide range of infections. Despite genetic diversity among these pathogens, common resistance strategies contribute to their emergence and persistence. These mechanisms include drug inactivation, reduced drug uptake, efflux pump activation, and potentially, BDCP-associated multi-drug resistance (Mancuso et al., 2021; Ramatla et al., 2024). However, the role of BDCPs in antibiotic resistance remains largely unexplored. Elucidating how BDCPs contribute to this phenomenon is a captivating area of research with the potential to revolutionize our understanding of bacterial resistance.

## Potential mechanisms of BDCPs in the development of antibiotic resistance

**Figure 4:**
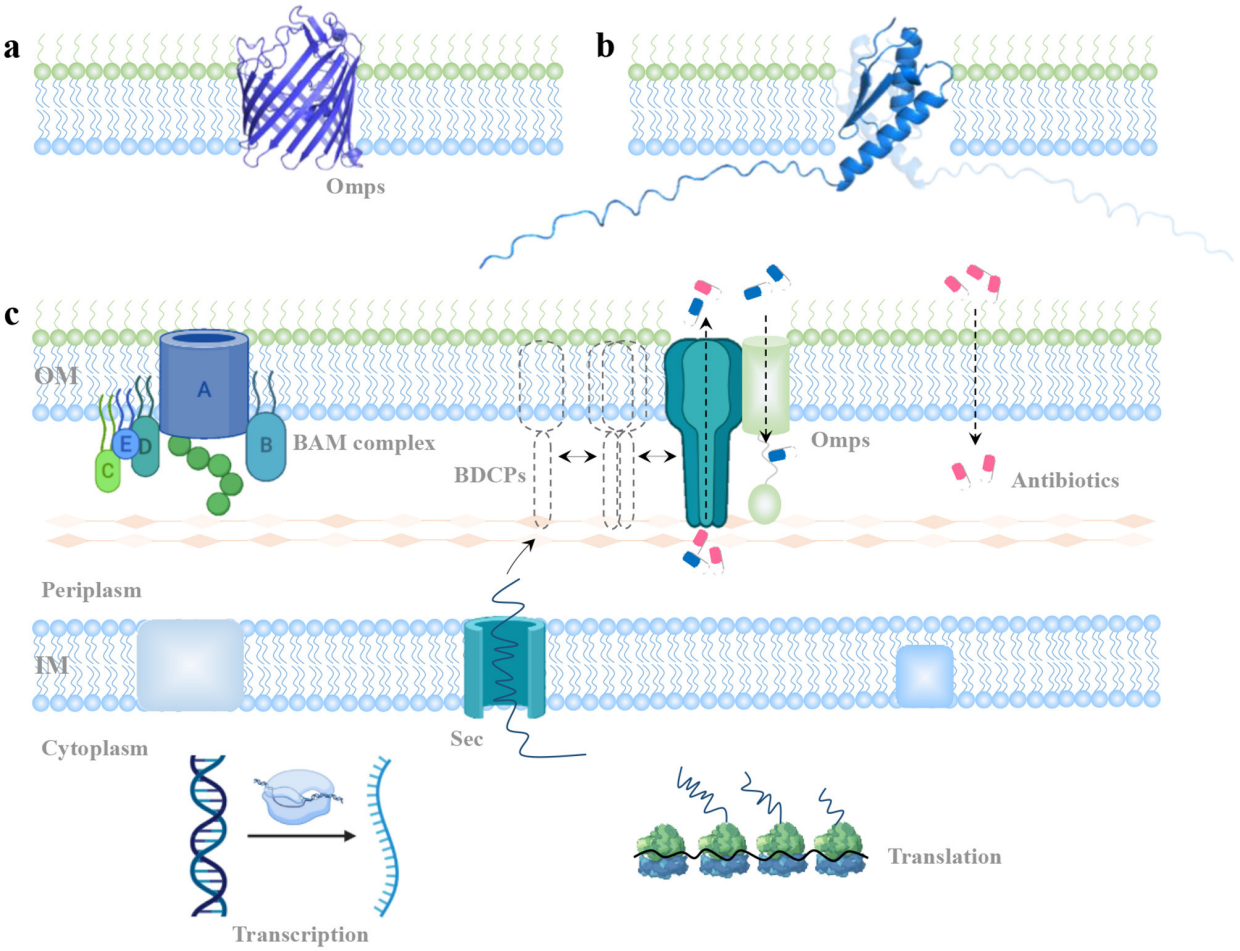
Potential mechanism of BDCPs-mediated antibiotic resistance. **(A)** View of OMPs monomer from the side. **(B)** View of BDCP with different special directions from *K. pneumoniae* embedded in the cell's outer membrane. **(C)** A model of BDCP-mediated development of antibiotic resistance. It describes the localization, oligomerization, and potential function of a typical BDCP throughout the outer membrane.

The outer membrane of Gram-negative bacteria (GNB) serves as a critical first line of defense, acting as a physical and mechanical barrier against external threats such as antibiotics (Hancock, 1997; Sun et al., 2022; Saxena et al., 2023). This selective barrier is composed of outer membrane proteins (OMPs). OMPs function as channels, regulating the passive or active uptake of small molecules. They typically consist of 8–24 β-strands arranged in a β-barrel structure. OMP classification is based on factors such as the number of subunits (monomer or trimer), and substrate specificity. Notably, general porins, a specific class of OMPs, allow for the non-specific diffusion of a range of small solutes up to 600 Da (Vergalli et al., 2020; Ude et al., 2021; Shen et al., 2023). In Enterobacteriaceae, for example, *E. coli* produces two major trimeric porins: OmpC and OmpF, which are utilized as prototypical structures to understand small-molecule permeability across bacterial membranes, in many cases (Masi et al., 2017). OmpF and OmpC orthologs are available from *K. aerogenes* (e.g., Omp35, Omp36), *K. pneumoniae* (e.g., OmpK35, OmpK36), and *E. cloacae* (e.g., OmpEc35, OmpEc36) (Pagès et al., 2008). The mechanisms of substrate transport facilitated by porins have been extensively studied. These channels form within the outer membrane, with each porin creating a central passage (Figure 4A). The amphipathic β-strands of porins, which are covalently connected by longer extracellular loops and short periplasmic turns, are usually cell surface exposed. The OMP channels, which allow small molecules across the plasma membrane are the major factors mediating antibiotic permeability and resistance.

Limited information on BDCP structures and functions necessitates careful inference when considering their potential role in antibiotic transport. Using a single-BON domain BDCP from *K. pneumoniae* strain as an example, its monomeric form appears unlikely to form a central channel architecture like typical OmpF and OmpC (Figure 4B). This observation necessitates further investigation into the potential assembly mechanisms of BDCPs and their role in antibiotic resistance development. Then a model was proposed to explain the emergence of BDCP-mediated antibiotic resistance (Figure 4C). In short, pathogenic bacteria harboring the BON domain gene typically express BDCPs under normal conditions or in response to external stress. The gene is transcribed into mRNA and translated into a nascent BDCP in the cytoplasm. For proteins containing a signal peptide, this sequence directs them to the Sec pathway for translocation across the inner membrane and into the periplasm. Here, transient polymerization and self-assembly may occur before the mature BDCP oligomers integrate into the outer membrane. These oligomers interact with peptidoglycan, spanning the periplasmic space. For BDCPs lacking a signal peptide, periplasmic chaperones or cytoplasmic enzymes might assist in translocation and proper folding within the cytoplasm (Goemans et al., 2014; Kim et al., 2021). The influx of antibiotic molecules into bacterial cells can occur *via* porins, bilayer diffusion, or self-uptake mechanisms. BDCP oligomers might play a role in antibiotic resistance through two possible, non-exclusive mechanisms: (1) Direct Efflux: BDCP oligomers could form channels in the outer membrane, directly facilitating the efflux of antibiotics from the cell. (2) Indirect Contribution: BDCPs might collaborate with other outer membrane proteins (OMPs) to maintain cell envelope integrity and stability. These roles could hinder antibiotic access to their targets within the bacteria, resulting in resistance development. Advanced structural biology techniques, such as NMR spectroscopy and cryo-EM, that have been widely used to unravel protein structures (Yip et al., 2020; Hu et al., 2021), could provide deeper insights into BDCP oligomerization in future research.

The fundamental facts for formulating this model are as follows: (1) bioinformatics show that BDCP is generally located in the membrane-bound part, and involved in molecular binding (such as protein and antibiotic binding), metabolic process, as well as response to stress (Sun et al., 2023); (2) some BDCPs are also capable of forming oligomers such as trimer (Sun et al., 2023) and decamer (Grinter et al., 2021) with a central pore architecture that is anchored to the periplasmic side of the outer membrane, and spans most of the periplasmic space; (3) the transmembrane oligomers contribute to the transport of antibiotic outside cells (Sun et al., 2023); (4) the expression of BDCP is upregulated in the presence of polymyxins, suggesting that BDCP plays an important role in supporting optimal outer membrane function (Henry et al., 2015; Cheah et al., 2016); (5) when localized to the cell division site, BDCP can interact with OmpA and share the outer membrane with the transmembrane divisome complex or the peptidoglycan (Samsudin et al., 2016; Wu et al., 2016).

Consequently, the bacterial cell envelope serves as a robust barrier against various antimicrobial agents, contributing to the persistence of bacterial infections in both environmental and clinical settings. To effectively combat these infections, new strategies are needed to overcome the formidable defenses of the outer membrane. This necessitates a deeper understanding of the key factors involved in outer membrane assembly and maintenance. In this context, investigating the roles of BON domain-containing proteins (BDCPs) offers valuable insights. Their potential involvement in outer membrane integrity and function warrants further study to elucidate their contribution to bacterial resistance mechanisms.

## Considerations and future directions

The rise of multidrug-resistant (MDR) Gram-negative pathogens poses a significant threat to global health. These bacteria exhibit alarming resistance to even last-resort antibiotics, partly due to their robust cell envelope. Unlike other bacteria, Gram-negative bacteria possess a unique outer membrane rich in diverse outer membrane proteins (OMPs) that contribute to their recalcitrance to antibiotics. Recent research has brought BON domain-containing proteins (BDCPs), a novel class of OMPs, to the forefront of antibiotic resistance discussions. However, a critical knowledge gap exists regarding the precise role of BDCPs in this process. Limited clinical data and a lack of in-depth understanding of these proteins hinder our ability to fully assess their contribution to bacterial resistance development. Further research on BDCPs is crucial to develop effective strategies to combat the emergence of widespread antibiotic resistance in pathogenic bacteria. Proactive measures are essential to stay ahead of this evolving threat.

This study sheds light on BON domain-containing proteins (BDCPs) by analyzing their prevalence in environmental microbes, summarizing their structural features, and proposing a potential mechanism for BDCP-mediated antibiotic resistance. Our findings reveal a surprisingly widespread distribution of BDCPs across diverse bacteria, including common pathogens. This ubiquity necessitates further investigation into their potential role in both intrinsic and acquired antibiotic resistance mechanisms, including interbacterial gene transfer. Furthermore, we model and predict the 3D structures of a panel of BDCPs from common pathogens (e.g., *E. coli, P. aeruginosa, K. pneumoniae*, and *A. baumannii*). Integrating distinct numbers of BON domains with or without the signal peptides among these bacteria indicated the diverse structural diversity and architecture of these BDCPs. The core BON domain is composed of two α-helix and three β-sheet, with an important role in antibiotic transport. Based on these findings and previous evidence, a potential BDCPs-mediated mechanism of antibiotic resistance development was proposed. The BDCPs need to form oligomer confirmations before they can interact with some other OMPs or function alone in the transport of antibiotics into the extracellular compartment. Generally, it appears that the oligomerization of these OMPs to form a central pore-like architecture is essential for the important function of the cell envelope that serves to protect bacteria from the unpredictable and often hostile environment, e.g., regulating membrane permeability to restrict entry of antibiotics into the cell. However, more studies are needed to fully understand the structure-function relationship of BDCPs. Here, we outline key areas for future exploration.

(1) It is necessary to further elucidate the precise role and function of BDCPs in the formation of antibiotic resistance in different pathogenic bacteria or common bacteria *via* gene transfer. For example, to what antibiotics can bacteria become resistant in the presence or absence of BDCPs? What is the structure-function relationship between compounds and antibiotic efflux? What is the extent of BDCPs-led antibiotic resistance in pathogens? What is the relationship between BDCP-mediated antibiotic transport and resistance in more clinical isolates? How does the BDCP gene transfer-based antibiotic resistance form? Is there an algorithm model that can be used to predict the BDCP gene transfer across bacteria species? At this point, multidisciplinary approaches integrating bacterial genetics, pathology, cell biology, biochemistry, physics, and bioinformatics are required to answer these questions.
(2) Some BDCPs have been identified from unculturable bacteria in soil and marine sediment metagenome. Investigation of the roles and functions of these BDCPs is also equally important. Proteins derived from metagenomics are often rare and novel species, with previously unidentified functions and activities. Collecting and analyzing BDCPs using metagenomic technology contributes to making up for the current deficiencies in microbial resources and possibly discovering new mechanisms in the development of antibiotic resistance.
(3) Versatile 3D structures of BDCPs inform us to explore the structure-based mechanism of BDCP during the formation of antibiotic resistance. Answering questions such as how these proteins oligomerize, how the oligomerization affects the function of BDCP, and how these oligomers are anchored into the cell membrane will be of great interest. It is expected that the structural biology that employs techniques such as X-ray crystallography, cryo-electron microscopy (cryo-EM), nuclear magnetic resonance (NMR) spectroscopy, and small angle X-ray scattering, and AI-based computing model strategy will be applied to address these challenges in the coming future.
(4) Understanding the effect of interactions between BDCPs and other cell membrane constituents such as some porins and BAM complex on the cell envelope permeability and the development of antibiotic resistance is considerable. Possibly weakening one of them, which could have a significant impact on the others, is likely to improve the effectiveness of a particular current antibiotic in the clinical practice and stimulate the development of new antimicrobial agents.
(5) Given different BDCPs have similar domain parts and share certain similarities in the protein sequences among different BDCP families, rapid identification and quantification methods of BDCP genes in specific clinical isolates could be developed using the PCR method based on the conservative motifs. This would greatly reduce the time and cost in the recognition of BDCP-containing pathogens in a complex system and understanding the resistance mechanism caused by BDCPs.
(6) Since BDCP is a consequence of within-host bacterial evolution that triggers specific responses and mutational adaptations, it also could be an inhibitory target to fight against antibiotic resistance. Similar approaches to the development of efflux pump or drug transporter inhibitors are applicable to BDCP. Compared with conventional and synthetic inhibitors, novel natural antimicrobial peptides represent a promising alternative to conventional antibiotics, which could impede the function of BDCPs by various mechanisms and restore the efficacy of antimicrobial agents for effective treatment in clinical applications. Although exploring specific AMP-BDCP interactions is beyond the scope of this review, future studies could delve into their unique effects on BDCPs and their role in combating antibiotic resistance.
