# Supplementary figures and images for: Unveiling the role of BON domain-containing proteins in antibiotic resistance
Source: Front Microbiol. 2025 Jan 7;15:1518045. doi: 10.3389/fmicb.2024.1518045 (PMC11747388; doi:10.3389/fmicb.2024.1518045)

**Graphical abstract**


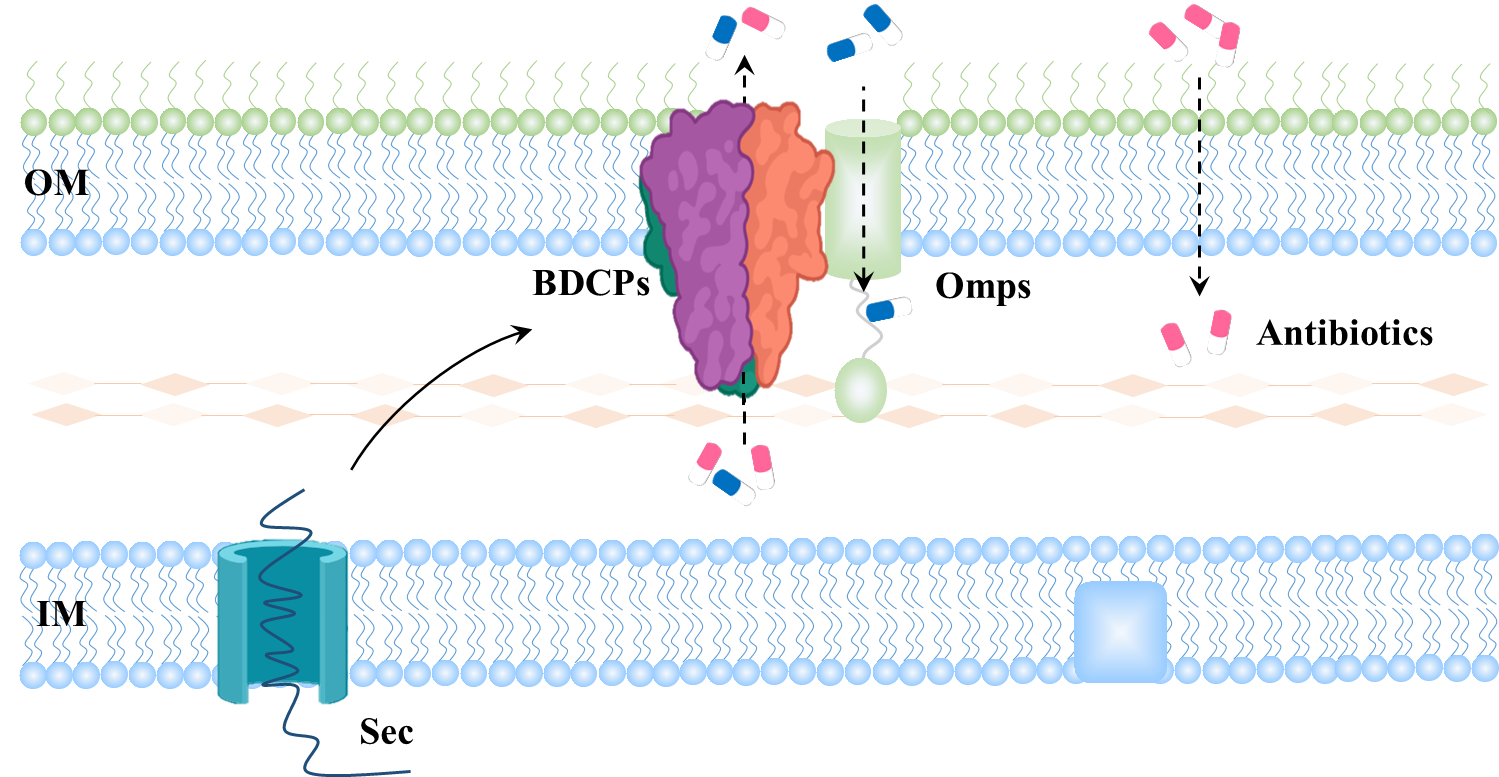

Supplement: Supplementary file 2 [file Table_2.docx]
