# Supplementary material for: Unveiling the role of BON domain-containing proteins in antibiotic resistance
Source: Front Microbiol. 2025 Jan 7;15:1518045. doi: 10.3389/fmicb.2024.1518045 (PMC11747388; doi:10.3389/fmicb.2024.1518045)
Supplement: Supplementary file 3 [file Table_3.docx]

**Unveiling the Role of BON domain-containing protein (BDCPs) in Antibiotic Resistance**

Shengwei Sun^a^*, Jinju Chen^b^*

^a^ School of Engineering Sciences in Chemistry, Biotechnology and Health, Department of Fibre and Polymer Technology, KTH Royal Institute of Technology, SE-100 44 Stockholm, Sweden

^b^ Department of Materials, Loughborough University, LE11 3TU, UK

^*^Corresponding author.

Shengwei Sun

Postal address: School of Engineering Sciences in Chemistry, Biotechnology and Health, Department of Fibre and Polymer Technology, KTH Royal Institute of Technology, SE-100 44 Stockholm, Sweden

E-mail: [ssw0929@163.com](mailto:ssw0929@163.com) ; [shengw@kth.se](mailto:shengw@kth.se)

Jinju Chen

Postal address: Department of Materials, Loughborough University, LE11 3TU, UK

E-mail: [jinju.chen82@gmail.com](mailto:jinju.chen82@gmail.com) ; [j.chen4@lboro.ac.uk](mailto:j.chen4@lboro.ac.uk);

**Supplementary protein sequences:**

>*Escherichia coli* (strain K12) - P64596 -Outer membrane lipoprotein DolP (191 AA)

MKALSPIAVLISALLLQGCVAAAVVGTAAVGTKAATDPRSVGTQVDDGTLEVRVNSALSKDEQIKKEARINVTAYQGKVLLVGQSPNAELSARAKQIAMGVDGANEVYNEIRQGQPIGLGEASNDTWITTKVRSQLLTSDLVKSSNVKVTTENGEVFLMGLVTEREAKAAADIASRVSGVKRVTTAFTFIK

> *Escherichia coli* (strain K12)- POAFH8 -Osmotically-inducible protein Y (201 AA)

MTMTRLKISKTLLAVMLTSAVATGSAYAENNAQTTNESAGQKVDSSMNKVGNFMDDSAITAKVKAALVDHDNIKSTDISVKTDQKVVTLSGFVESQAQAEEAVKVAKGVEGVTSVSDKLHVRDAKEGSVKGYAGDTATTSEIKAKLLADDIVPSRHVKVETTDGVVQLSGTVDSQAQSDRAESIAKAVDGVKSVKNDLKTK

> *Pseudomonas aeruginosa*- A0A3M5EB07- BON domain-containing protein (181 AA)

ALSLALGGCSSFLSATRDKPIDDDRGTRTIGSKIDDSLIETKAAVNIAKADPALDKDSHIVVVSYNGIVLIAGQTPRADLKSKAEQAARTVQKVKNVHNELQVTSPSSLLARNNDAWITTKLKTQMLSDPNVPSSRIKVVTENGIVYMMGLVNQQEAAQAVRVAQGVDGVQKIVKLFEYIN

> *Klebsiella pneumoniae* IS43- W1DIA8- BON domain-containing protein (104 AA)

MKWFKAIPAFCATLLVTTALAGCAGSATKESTGGYIDDTVVTTKVKTALFNDKDIKSSEISVQTFKGRVQLSGFVSSAESAKRAVEVTRRVQGVRMVENDLRIK

> *Acinetobacter baumannii* WM99c- A0A385EU76- BON domain-containing protein (235 AA)

MLKRIAVTALCVASLSGCASFISGGTGTAPVGTDSGVRSLGQVFIDSSIKRTANINLYKLDQRFKQSRINIESFHSTVLLTGQVPDPYLKQLAEDNVKAMSDVKAVHNYITVGNKVSYNTIMQDAGVTANTRALLMKAPVVSDSKVLVHTEDGVLYVMGRLNTAEINDLNNVLQNVGNVTKIVTLIDNIDLAPAPAASTASATTTPVINNVLAQPTVQTPVAIDPDQTDPASSAQ

> *Burkholderia multivorans*- A0A2S9LL40- BON domain-containing protein (216 AA)

MKSDSELKKDVEQELEWDPSINAVRIGVEVHERIVTLTGHVGSYLEKVAVRKAVERIEGVRGIVLELGVQTDDGKRRSDEDIAIAARSLLMWDAGLGEQAVQVTVENGCVTLSGEVMWSYQMQEAERAVERLRGVTRIINEIHVRPHPTHSDIAGKIQAALIRHATEEAAHVGITVQDGCVTLTGQVGSLSERRLAFDAAWSAPGVRDVVDQLTVA

> *Vibrio parahaemolyticus*- A0A7Y0XGJ4- BON domain-containing protein (227 AA)

MIRSLVILCTILSLSGCAGLFIAGAATTANIVTDTRTTKQIWQDNNIEFEVAAIGNKEPFKGHVRVVASSYNGTVVLMGQAPTQDLINQIEQRARAIDGVKTIHNQIKAKEPLSVTQISNDSWITTKVKSALLTDSDLNGVKVKVITEDGDVFLFGYVTAQQGERATEISRNISGVKQVIKGFQYGDAEPVKVDLPSDGVTTQTQTQTQTQTQDDSDPDVETFTISD

> *Legionella pneumophila*- A0A128Y4Q3- BON domain-containing protein (103 AA)

MLKCLSSVIIVFLILFTAGCQTNTVSNMFIPSNPSGMTLAQSVKDSLMESNDPVINQIHVESNQNVVILSGYVKKIRQSDIAEQIARQVQGVQSVENRIIVRP

> *Burkholderia cenocepacia*- A0A6B2MM63- BON domain-containing protein (283 AA)

MQRRYPGRSGERGGPPDWHERNERAYRGAGERGIPDDPARWPEEDYESAYRRFASEDVGPEDWGSEWTERAARPVDPRRAAADPDWPGGARQPARTPERGYGDPGRRAEGREGRYGGERPEFRGQHRMGWYGGERDPGDPRHGGSGPDLSRFGRDTEREALRHRRGPKGYTRSDERIREDVCERLAHALEIDVSDVTVQVRDGRVELDGTVPARWMKHDIEDLADGCMGVQDVENRVRVRREGEHDTGMVLHPDQRTVTPTQPAARDPEPGTVVRGREREPRH

> *Burkholderia pseudomallei* (strain K96243)- Q63IZ2- BON domain-containing protein (216 AA)

MKTDRQIKQEVEDELTGDPMIDVAHFDVDVAGHIVTLTGHPSSYAEKLAAEKAANRVAGVRAVVVDVQVRLPSDDVRTDEAIADAVRSTLHWTVGLHDAAVHVQVEAGWVTLSGQVDWPYQSHVAARAISQMRGVTGVTNQIEVLGDISADEIAGGIRRAMQRHAEREANHIDVTVKDGTVTLAGKVGSYAERAVARGAAWSARGVRAVVDDLVVE

> *Sinorhizobium meliloti* (strain SM11)- Q1WL90- BON domain-containing protein (225 AA)

MDTDVRLRQDILDELQYEPSIDAANIGVAVEDGIVTLTGHVRSYAEKHAAERIAERVNGVRAIAEEIDVRLPEHKKTADDEIAGRVLKILAWGAAISDPEDITVKVEKGFVTLEGTVDWHFQRSAAENSVRVLTGVTGIDNQLRIRPRMNVVDVRHGIREALKRNAETEAENIDVEVSGSHVILHGKVQSLRARALAERAAWSAPGVTAVEDRLRIEDARVALGT

> *Salmonella enterica* (*Salmonella choleraesuis*)- A0A403T4X8- BON domain-containing protein (157 AA)

MTARFCGENQKMKHLPGILSFFFITLLSGCAPAAVLITTVTARTAADPRSTGRQIDDGTLALRVSHTISEAGLSPQARVTATVYQGDVLLTGEVPDDATRQAATKAVLSVAGVRHTSSRIFFNGRAEFSLWSIPSTGTCLPRLCFTRSERGIYPGPG

**Note**: Amino acid residues in red indicate an identified signal peptide.


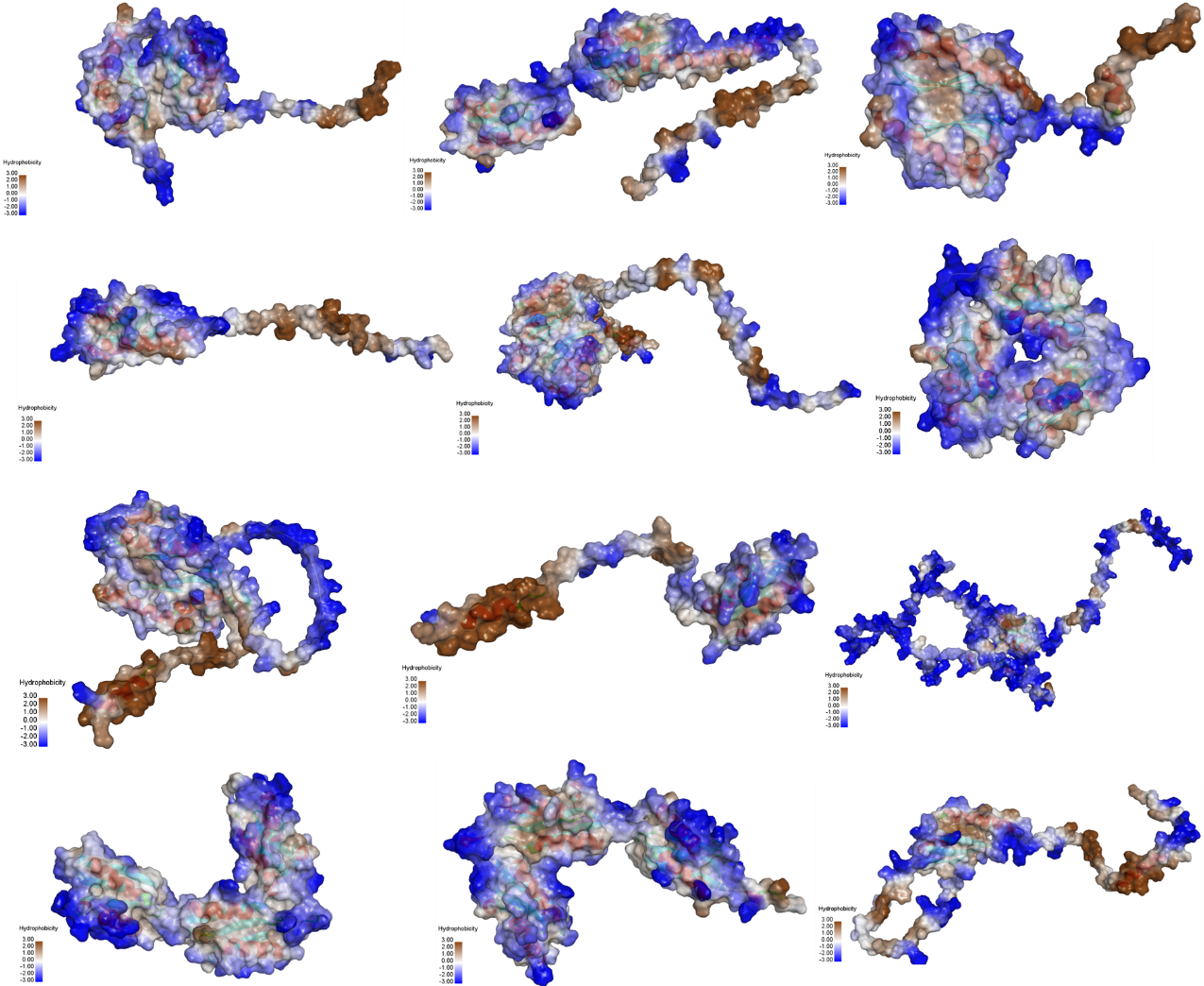


**Figure S1** Hydrophobicity analysis among these BDCPs. Tan areas indicate increased hydrophobicity, while blue areas indicate decreased hydrophobicity. The hydrophobicity score is shown in the lower left corner.
